# Effects of Combined Physical and Cognitive Stimulation on Go/No-Go Performance in Older Women: A Pilot and Feasibility Study

**DOI:** 10.3390/jintelligence14090205

**Published:** 2026-09-01

**Authors:** Mauricio Barramuño-Medina, Pablo Valdés-Badilla, Pablo Aravena-Sagardia, Jordan Hernandez-Martínez, Edgar Vásquez-Carrasco, Esteban Fariña-Hermosilla, Wilson Pastén-Hidalgo, Germán Gálvez-García

**Affiliations:** 1 Kinesiology Program, Faculty of Health Sciences, Universidad Autónoma de Chile, Temuco 4780000, Chile; mauricio.barramuno@uautonoma.cl; 2 Department of Physical Activity Sciences, Faculty of Education Sciences, Universidad Católica del Maule, Talca 3460000, Chile; 3 Sport Trainer Program, Faculty of Life Sciences, Universidad Viña del Mar, Viña del Mar 2520000, Chile; 4 Physical Education Pedagogy, Faculty of Education, Universidad Autónoma de Chile, Temuco 4780000, Chile; pablo.aravena@uautonoma.cl; 5 Department of Physical Activity Sciences, Universidad de Los Lagos, Osorno 5290000, Chile; jordan.hernandez@ulagos.cl; 6 Department of Education, Faculty of Humanities, Universidad de la Serena, La Serena 1700000, Chile; 7 Occupational Therapy School, Faculty of Psychology, Universidad de Talca, Talca 3465548, Chile; edgar.vasquez@utalca.cl; 8 Centro de Investigación en Ciencias Cognitivas, Faculty of Psychology, Universidad de Talca, Talca 3465548, Chile; 9 VITALIS Longevity Center, Universidad de Talca, Talca 3465548, Chile; 10 Department of Rehabilitation Sciences, Faculty of Medicine, Universidad de La Frontera, Temuco 4780000, Chile; esteban.farina@ufrontera.cl; 11 Department of Kinesiology, Faculty of Health Sciences, Universidad de Atacama, Copiapó 1530000, Chile; 12 Department of Experimental Psychology, Psychobiology and Behavioral Sciences Methodology, Universidad de Salamanca, 37005 Salamanca, Spain; germangalvezgarcia@usal.es

**Keywords:** older women, multicomponent training, cognitive stimulation, inhibitory control, executive function

## Abstract

This study aimed to examine within-subject changes associated with a 12-week multicomponent training program combined with cognitive stimulation in cognitive–motor performance in older women, and to evaluate changes in cognitive status and health-related quality of life (HRQoL). Thirty-three participants aged 70.24 (4.32) years were assessed at baseline, 6 weeks, and 12 weeks. Cognitive–motor performance was evaluated using a Go/No-Go task, including reaction time (RT), omission errors, and commission errors. Cognitive status was assessed using the Memory, Fluency, and Orientation (MEFO) score, and HRQoL was evaluated with the 36-Item Short Form Health Survey (SF-36). Mixed-effects models showed a significant reduction in RT (Δ = 51.5 ms, *p* < 0.001; d = 0.50) and omission errors (OR = 0.04, *p* < 0.001), and an increase in commission errors (OR = 2.06, *p* = 0.008). Cognitive status improved significantly (Δ = 0.45, *p* = 0.021; d = 0.34), whereas HRQoL improved only in the general health dimension (Δ = 6.82, *p* = 0.004; d = 0.53). Overall, the intervention was associated with a reorganization of Go/No-Go performance, characterized by faster responding, but increased commission errors, indicating no improvement in motor inhibition. These findings suggest that this multidomain approach is feasible and warrants evaluation in randomized controlled trials.

## 1. Introduction

Aging is a progressive process characterized by gradual declines in both physiological and cognitive systems, ultimately affecting health, functional independence, and health-related quality of life (HRQoL) (Vints et al., 2024). These age-related changes are influenced by multiple interacting factors, including physical inactivity, comorbidities, and lifestyle behaviors, which can accelerate functional deterioration over time (Falck et al., 2017). In this context, aging is commonly associated with changes in body composition and reductions in physical capacities, including muscle strength, mobility, and postural balance (McKinnon et al., 2017). Cognitive decline also represents a central feature of this process, with important implications for daily functioning and rehabilitation outcomes.

In line with these changes, cognitive aging is marked by impairments in memory, processing speed, and executive functions (Stillman et al., 2020). Within this framework, cognitive control enables the regulation of goal-directed behavior, including the ability to inhibit prepotent responses (Diamond, 2013). Motor inhibition, as a specific domain of inhibitory control, refers to the ability to suppress an inappropriate or prepotent motor response when the context requires withholding an action (Diamond, 2013; Verbruggen & Logan, 2008), hereafter referred to as motor inhibition (MI).

From a functional and rehabilitation perspective, MI is particularly relevant as many daily activities require older people not only to initiate movements efficiently but also to stop, delay, or modify actions in response to environmental demands (Diamond, 2013). In Go/No-Go paradigms, MI is primarily indexed by commission errors, that is, incorrectly executed responses to No-Go stimuli. By contrast, reaction time (RT) on correct Go trials reflects response speed or cognitive–motor processing efficiency, whereas omission errors, defined as failures to respond to Go stimuli, are more closely related to attention, vigilance, or task engagement (Vernooij & Smits, 2012). Therefore, the joint interpretation of RT, omission errors, and commission errors allows characterization of the balance between response activation and response suppression, rather than treating all three indicators as equivalent measures of inhibition (Diamond, 2013; Vernooij & Smits, 2012).

Empirical evidence using Go/No-Go and stop-signal paradigms indicates that aging is associated with reduced inhibitory efficiency, typically reflected in slower response times and increased difficulty suppressing inappropriate responses (Verbruggen & Logan, 2008; Wessel, 2018). Importantly, these changes reflect not only a generalized deficit, but also strategic adaptations, as older people often prioritize accuracy over speed, adopting more conservative response patterns. Accordingly, performance in inhibitory tasks reflects both inhibitory processes and speed–accuracy trade-offs rather than a unitary decline in inhibitory capacity (Heitz, 2014). Declines in MI are particularly relevant in older people, as they have been associated with reduced ability to suppress inappropriate actions, increased fall risk, and impaired decision-making in daily life (Healey et al., 2026). These changes may occur even in the absence of clinical neurodegenerative conditions, yet still contribute to reduced independence and diminished HRQoL (Sofi et al., 2011). At the neural level, aging is associated with structural and functional alterations, including brain atrophy, reduced neural efficiency, and decreased gray- and white-matter integrity, which are closely linked to declines in higher-order cognitive processes (Stillman et al., 2020; Lee & Kim, 2022).

Importantly, the intervention was not designed to train inhibitory control or Go/No-Go performance directly, but to target broader cognitive–motor processes, including processing speed, attentional control, and action execution. Accordingly, changes in Go/No-Go performance should be interpreted as reflecting modifications in the balance between response activation and suppression, rather than a direct enhancement of inhibitory capacity.

To further examine these issues, the present study aimed to assess within-subject changes in Go/No-Go performance associated with a 12-week MCT program combined with cognitive stimulation activities in older women. MI was operationalized as commission errors, while RT and omission errors were considered complementary indicators of cognitive–motor efficiency and task engagement. A secondary aim was to examine changes in cognitive status and HRQoL. It was hypothesized that the combined intervention would be associated with faster responses and fewer omission errors, reflecting improved cognitive–motor efficiency. Given the potential influence of speed–accuracy trade-off effects, changes in commission errors were examined to determine whether faster responding was accompanied by improved or reduced response suppression. Additionally, improvements in cognitive status and HRQoL were expected.

## 2. Materials and Methods

### 2.1. Study Design

This study followed a pre-experimental repeated-measures design and was conducted as a pilot and feasibility study for a larger randomized controlled trial registered at ClinicalTrials.gov (Identifier: NCT05275140). The primary aim was to evaluate the feasibility and preliminary within-subject changes associated with the intervention to inform the design and implementation of the subsequent trial. A single-group pre-experimental study with repeated measures over time was conducted in accordance with the TREND guidelines for nonrandomized intervention studies (Haynes et al., 2021), following a single-blind design in which outcome assessors were blinded to the intervention phase. Primary outcome (MI) measures were assessed at baseline, 6 weeks, and 12 weeks, whereas secondary outcomes (cognitive status and HRQoL) were assessed at baseline and 12 weeks. This design enabled the assessment of within-subject changes over time. The intervention lasted 12 weeks and comprised 36 sessions. Sessions were held three times per week (Monday, Wednesday, and Friday), each lasting 60 min. All assessments were conducted under standardized laboratory conditions by trained evaluators. Throughout the intervention period, participants did not report injuries, and no pain was experienced either before the assessments or during the training sessions. The present pilot study was specifically designed to inform the methodological planning of the subsequent randomized controlled trial, including recruitment procedures, intervention feasibility, and outcome selection.

### 2.2. Participants

**Figure 1:**
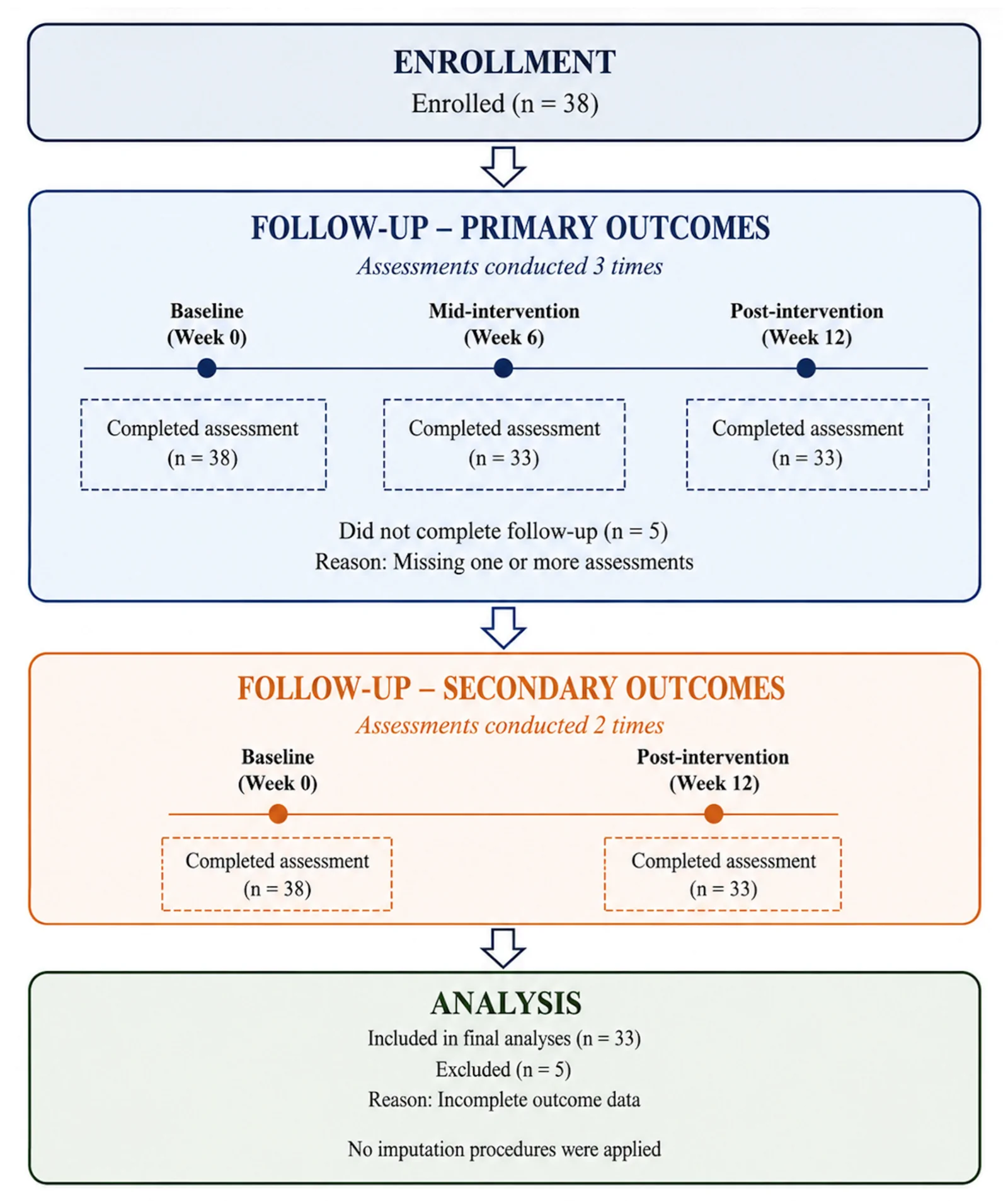
Participant flow diagram.

A total of 38 participants were initially enrolled. Thirty-three completed assessments at all time points (baseline, 6 weeks, and 12 weeks) and were retained for the final analysis, whereas five participants were excluded due to incomplete outcome data. No imputation procedures were applied. Overall adherence to the intervention was 86.8%. No intervention-related adverse events were reported during the 36 sessions or throughout the assessment period. The flow of participants throughout the study is presented in Figure 1. Participants were recruited via convenience sampling from community-dwelling, physically inactive older people enrolled in programs affiliated with the Pedro Pastor Family Health Center in Temuco, Chile. Recruitment and data collection were conducted between March and June 2025 at the same facility. Sample size estimation was conducted based on a repeated-measures design with three assessment time points. Assuming a small-to-moderate effect size (f = 0.15), derived from previous meta-analytic evidence (Northey et al., 2018; Lin et al., 2025), an alpha level of 0.05, and a statistical power of 80%, the required sample size was estimated to be approximately 28 to 36 participants. Inclusion criteria were: (i) women with age between 65 and 80 years; (ii) ability to understand and follow simple instructions; (iii) functional independence to perform basic activities of daily living; and (iv) commitment to attend at least 85% of the intervention sessions and complete assessments at all time points. Exclusion criteria included: (i) presence of musculoskeletal or neurological conditions that could limit participation in physical activity; (ii) ongoing rehabilitation or medical restrictions contraindicating physical activity; (iii) current participation in a structured exercise program; and (iv) inability to complete the assessment procedures. All participants provided written informed consent prior to participation. The study protocol was approved by the Scientific Ethics Committee of the Universidad Católica del Maule, Chile (No. 29-2022), and was conducted in accordance with the Declaration of Helsinki.

### 2.3. Intervention

Participants completed a 12-week MCT program with three sessions per week, each lasting 60 min, divided into 10 min of warm-up, 40 min of the main phase, and 10 min of cool-down. All sessions were supervised by an instructor with a master’s degree in Gerontology to ensure correct exercise execution and optimize adherence to the program. The MCT included a combination of strength, balance, coordination, and aerobic exercises, which were progressively adjusted in terms of intensity and complexity throughout the intervention period. Exercises were performed using body-weight and external implements such as dumbbells, kettlebells, poles, and medicine balls. Training intensity was prescribed and monitored using the rating of perceived exertion (RPE) scale (0–10), targeting a moderate-to-vigorous intensity corresponding to RPE values of 5–7 throughout the intervention. The training volume was progressively periodized from three sets of 10 repetitions (weeks 1–4) to four sets of 10 repetitions (weeks 5–8), and finally to four sets of 12 repetitions (weeks 9–12). In contrast, a constant 2 min rest interval between sets was maintained throughout the intervention. Each session began with a warm-up that included joint mobility exercises and low-intensity aerobic activity (e.g., treadmill walking, stationary cycling, or instructor-guided activation exercises). The main phase consisted of six MCT exercises targeting the major muscle groups of the upper limbs (biceps brachii, triceps brachii, deltoids, and latissimus dorsi) and lower limbs (quadriceps femoris, hamstrings, gluteal muscles, and the gastrocnemius). During the main phase, participants engaged in rest intervals between physical exercise sets and in cognitively oriented activities categorized as cognitively stimulating activities or structured cognitive training, which were alternated across sessions. These activities were standardized and systematically alternated across sessions, including leisure-based tasks (e.g., crossword puzzles, word searches, reading, and mindfulness) and structured exercises targeting memory, attention, and processing speed (Sprague et al., 2019). Each cognitive activity was performed for a fixed duration of 2 min during the recovery periods between exercise sets. Session A and Session B were implemented in a fixed alternating sequence (A–B–A–B) throughout the 12-week intervention. Cognitive demands remained constant across the intervention; however, different versions of the same cognitive tasks were used to maintain stimulus novelty while preserving comparable cognitive demands. In contrast, the motor component of Session B progressed from upper-limb movements during the initial weeks to whole-body movements during the later stages of the intervention. All intervention sessions were delivered by the same instructor using standardized instructions. The cool-down was to be completed through exercises for both static and dynamic flexibility. Details of these activities and their periodization are presented in Table 1.

### 2.4. Primary Outcomes

#### Motor Inhibition (MI)

MI was assessed using a computerized Go/No-Go task, consistent with the operational definition provided in the Introduction. The task was administered individually in a quiet, temperature-controlled laboratory environment with standardized lighting conditions. Participants wore noise-canceling headphones to minimize external distractions. The task was implemented using OpenSesame software (version 4.1.6; Mathôt et al., 2012) and presented on a monitor positioned approximately 60 cm from the participant. Visual stimuli consisted of colored circles (blue and yellow) displayed randomly within a fixed central area on the screen. Each stimulus was presented for 2000 ms, during which participants were required to respond. Participants were instructed to respond as quickly as possible to Go stimuli by pressing the space bar and to withhold their response when a No-Go stimulus appeared. The stimulus–response mapping was counterbalanced across participants to control for potential color-related biases. Feedback for incorrect responses was provided through a brief visual signal displayed at the center of the screen. The task comprised 160 trials, including 128 Go trials and 32 No-Go trials (80/20 proportion) to establish a prepotent response tendency. Trials were organized into 8 blocks of 20 trials with short rest intervals between them to reduce fatigue. The intertrial interval varied randomly between 1000 and 1500 ms to minimize anticipatory responses and temporal predictability (Wessel, 2018; Barry et al., 2018). RT was calculated for correct Go trials as the latency between stimulus onset and response execution. Accuracy was quantified using omission errors (failures to respond to Go stimuli) and commission errors (responses to No-Go stimuli), both treated as binary outcomes.

### 2.5. Secondary Outcomes

#### 2.5.1. Cognitive Status

Cognitive status was evaluated using the memory, phonetic fluency, and temporal–spatial orientation (MEFO, by its Spanish acronym) survey, a brief and clinically validated screening instrument designed to assess global cognitive status in older people (Delgado Derio et al., 2013). The MEFO comprises three core domains: episodic memory, assessed through delayed recall tasks; phonetic fluency, typically evaluated via category-based word generation; and temporal–spatial orientation, which examines awareness of time-related information. These components are integrated into a composite score that reflects overall cognitive status. Higher scores indicate better cognitive status.

#### 2.5.2. Health-Related Quality of Life (HRQoL)

HRQoL was assessed using the 36-Item Short Form Health Survey (SF-36), a widely validated instrument for evaluating perceived health status across multiple domains (Vilagut et al., 2005). The SF-36 comprises eight subscales: physical function, physical role, body pain, general health, vitality, social function, emotional role, and mental health. The SF-36 has demonstrated adequate reliability and validity in older people (Vilagut et al., 2005). To ensure consistency, all evaluations were conducted under standardized conditions using the same equipment and trained personnel across sessions.

### 2.6. Covariates

#### 2.6.1. Age (Years)

Age was recorded in years during a structured interview conducted at baseline. Age was included as a continuous covariate given its well-established influence on cognitive and motor performance in older people (Stillman et al., 2020; Northey et al., 2018).

#### 2.6.2. Body Mass Index (BMI)

Body weight was measured using a digital scale (Seca 769, seca GmbH & Co. KG, Hamburg, Germany; accuracy 0.1 kg), and bipedal height was assessed with a stadiometer (Seca 220, seca GmbH & Co. KG, Hamburg, Germany; accuracy 0.1 cm). All measurements were performed in accordance with the guidelines of the International Society for the Advancement of Kinanthropometry (ISAK) (Marfell-Jones et al., 2012) by a certified level II anthropometrist. BMI was calculated as body weight (kg) divided by the square of height (m^2^).

### 2.7. Statistical Analysis

Analysis was conducted in R (version 4.5.1) using RStudio (version 2025.05.1+513). Continuous outcomes were analyzed using linear mixed-effects models (LMMs), whereas binary outcomes were analyzed using generalized linear mixed-effects models (GLMMs) with a binomial distribution and logit link function. All models were fitted using the lme4 package (version 1.1-35.1; Bates et al., 2024). Descriptive statistics are presented as mean (standard deviation) or percentages, as appropriate. In all models, subject was included as a random intercept to account for repeated measures. For RT, only correct trials were analyzed. Anticipatory responses (<150 ms) were excluded prior to analysis. Competing models, including session alone and in combination with covariates (age, BMI, and cognitive status score), as well as interaction terms, were compared using the Akaike Information Criterion corrected for small samples (AICc). The model with the lowest AICc was retained, with preference for parsimony when ΔAICc < 2. Fixed effects were evaluated using Type III analysis of variance with Satterthwaite’s approximation, and model fit was quantified using marginal and conditional R^2^. Omission and commission errors were analyzed by GLMMs, and results are reported as odds ratios (OR) with 95% confidence intervals. Cognitive status was analyzed at both the total-score and domain levels, with session, domain, and their interaction included as fixed effects. HRQoL was analyzed across the eight SF-36 subscales. Estimated marginal means (EMMs) were computed for all models, and pairwise comparisons were adjusted using the Bonferroni correction. Effect sizes were calculated for statistically significant effects, including standardized mean differences for continuous outcomes and OR for binary outcomes. Statistical significance was set at *p* < 0.05.

## 3. Results

A total of 33 older women were included in the study. At baseline, participants had a mean age of 70.24 (4.32) years (range: 65–80), a body weight of 71.57 (13.91) kg (range: 50.0–99.0), and a bipedal height of 151.06 (8.21) cm (range: 141–173). The mean BMI was 31.21 (5.49) kg/m^2^ (range: 22.8–43.4). Baseline cognitive status, assessed using the MEFO, was 11.48 (1.60) (range: 8–13).

Model comparison indicated that the session-only and BMI-adjusted models showed comparable support (ΔAICc < 2). Consistent with the prespecified model selection criterion favoring parsimony when competing models differed by less than two AICc units, the simpler session-only model was retained for subsequent analyses.

### 3.1. Feasibility Outcomes

A total of 38 older women were enrolled in the study, of whom 33 completed all study assessments, corresponding to an 86.8% retention rate. Five participants were excluded from the final analyses because they did not complete all study assessments. These exclusions were unrelated to the intervention itself, and no intervention-related adverse events were reported during the study. Overall adherence to the intervention was high, supporting the feasibility and safety of implementing the multidomain intervention in this population.

### 3.2. Motor Inhibition

#### 3.2.1. Reaction Time

**Figure 2:**
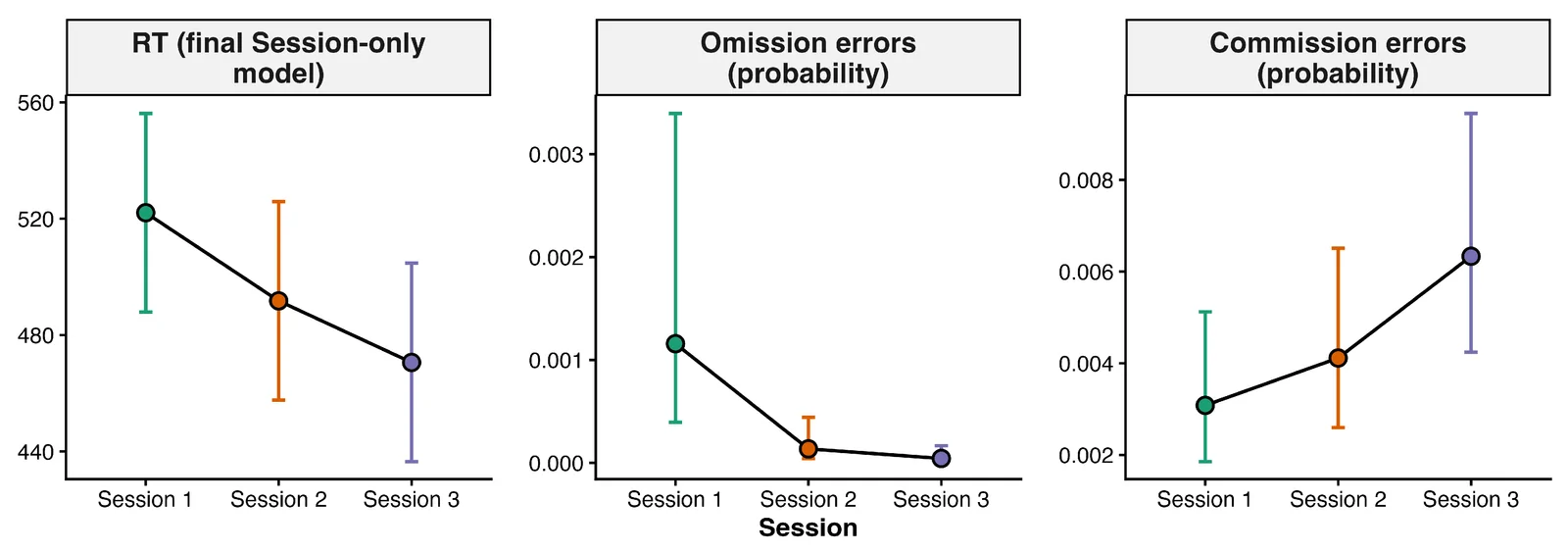
Estimated marginal means (95% confidence intervals) for reaction time (RT) across sessions derived from the linear mixed-effects model.

Fixed effects from the final linear mixed-effects model are presented in Table 2. A significant effect of session on RT was observed (F_(2, 11,721.38)_ = 154.49, *p* < 0.001). The model explained a small proportion of variance attributable to the fixed effects (marginal R^2^ = 0.016). In contrast, the conditional R^2^ was 0.380, indicating that much of the remaining variance was accounted for by between-participant differences. Estimated marginal means showed a progressive reduction in RT across sessions (see Figure 2), corresponding to an overall reduction of approximately 10% from Session 1 to Session 3.

Bonferroni-adjusted pairwise comparisons revealed significant reductions in RT between Session 1 and Session 2 (Δ = 30.3 ms, *p* < 0.001), Session 1 and Session 3 (Δ = 51.5 ms, *p* < 0.001), and Session 2 and Session 3 (Δ = 21.2 ms, *p* < 0.001). At the individual level, 76% of participants showed a reduction in RT from Session 1 to Session 3. The reduction in RT from Session 1 to Session 3 corresponded to a moderate effect size (Cohen’s d = 0.50), whereas the change between Session 1 and Session 2 was small (Cohen’s d = 0.35).

#### 3.2.2. Omission and Commission Errors

A significant effect of session was observed for omission errors. The likelihood of omission errors was significantly reduced in Session 2 (OR = 0.12, 95% CI: 0.07–0.20, *p* < 0.001) and Session 3 (OR = 0.04, 95% CI: 0.02–0.09, *p* < 0.001) compared with Session 1. Pairwise comparisons indicated lower omission error probability in both Session 2 and Session 3 relative to Session 1 (both *p* < 0.001), with no significant difference between Session 2 and Session 3 (*p* = 0.053).

For commission errors, a significant effect of session was also found. The likelihood of commission errors was significantly higher in Session 3 compared with Session 1 (OR = 2.06, 95% CI: 1.21–3.52, *p* = 0.008), whereas no significant difference was observed in Session 2 (OR = 1.34, 95% CI: 0.75–2.39, *p* = 0.327). Pairwise comparisons showed a higher probability of commission errors in Session 3 compared with Session 1 (*p* = 0.024), with no differences between Session 1 and Session 2 (*p* = 0.980) or between Session 2 and Session 3 (*p* = 0.244).

### 3.3. Cognitive Status

**Figure 3:**
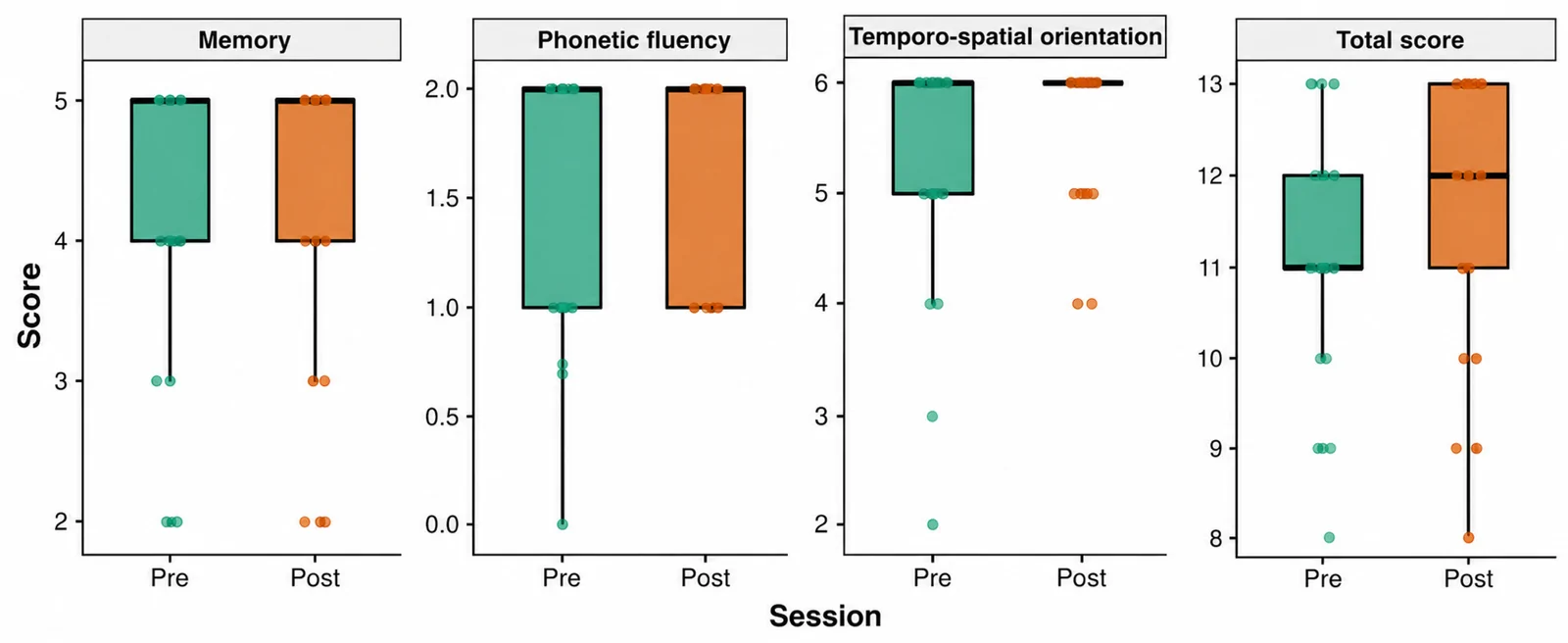
Changes in MEFO subdimensions from pre- to post-intervention. Boxplots represent the distribution of scores for each cognitive domain, including phonetic fluency, memory, temporo-spatial orientation, and total score, at Pre and Post assessments. The central line within each box indicates the median, the box represents the interquartile range (IQR), and whiskers extend to the most extreme values within 1.5 × IQR. Individual data points are displayed to illustrate within-subject variability.

Estimated marginal means for the MEFO total score and its subdimensions are presented in Figure 3. Bonferroni-adjusted pairwise comparisons revealed a significant increase only in the total MEFO score (Δ = 0.45, *p* = 0.021). In contrast, changes in phonetic fluency (Δ = 0.09, *p* = 0.642), memory (Δ = 0.06, *p* = 0.756), and temporo-spatial orientation (Δ = 0.27, *p* = 0.163) were not statistically significant. The increase in the total MEFO score corresponded to a small-to-moderate effect size (Cohen’s d = 0.34), whereas the individual subdomains showed small effect sizes (temporo-spatial orientation: d = 0.27; phonetic fluency: d = 0.16; memory: d = 0.05).

### 3.4. Health-Related Quality of Life (HRQoL)

**Figure 4:**
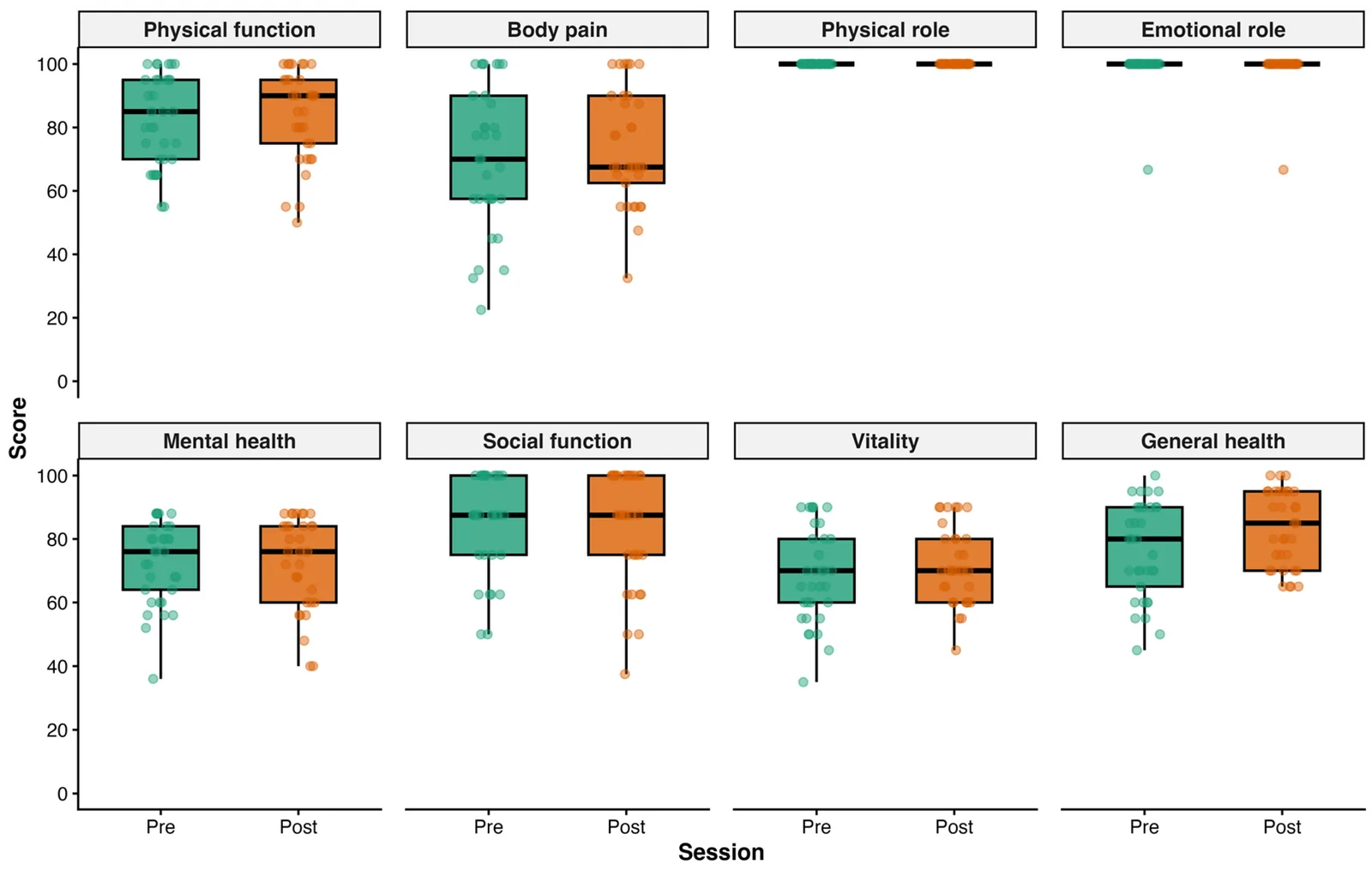
Changes in SF-36 domain scores from pre- to post-intervention. Values are presented as estimated marginal means (points) with 95% confidence intervals (error bars) derived from linear mixed-effects models. Higher scores indicate better health-related quality of life. The central line within each box indicates the median, the box represents the interquartile range (IQR), and whiskers extend to the most extreme values within 1.5 × IQR. Individual data points are displayed to illustrate within-subject variability.

Estimated marginal means for all SF-36 domains are presented in Figure 4. A significant increase was observed only in the general health domain (Δ = 6.82 points, *p* = 0.004; Cohen’s d = 0.53). No statistically significant changes were detected in the remaining HRQoL domains (all *p* > 0.05). Small, non-significant effect sizes were observed for physical function (d = 0.16), body pain (d = 0.14), vitality (d = 0.20), mental health (d = −0.01), and social function (d = −0.09). Emotional role remained unchanged, whereas physical role exhibited a ceiling effect, with identical scores across all participants at both assessment time points.

## 4. Discussion

The present study examined within-subject changes associated with a 12-week MCT program, combined with cognitive stimulation activities, in MI, operationalized as RT, omission errors, and commission errors, in older women. A secondary aim was to examine changes in cognitive status and HRQoL across the study period. The main findings indicate a systematic shift in Go/No-Go performance across sessions, characterized by faster RT, fewer omission errors, and increased commission errors. This pattern is consistent with a speed–accuracy trade-off, rather than a uniform improvement in inhibitory control. In parallel, cognitive status improved significantly over time, whereas HRQoL showed changes restricted to the general health dimension.

Importantly, this pattern should be interpreted in light of the conceptual distinction between performance components. While commission errors represent the primary behavioral index of MI, RT and omission errors reflect complementary processes related to response speed, attentional engagement, and cognitive–motor efficiency. Notably, commission errors, the primary index of motor inhibition, did not improve. Accordingly, the observed pattern does not support a direct enhancement of motor inhibition. Instead, it should be interpreted as a reorganization of Go/No-Go performance and response strategy, in which participants adopted faster response strategies accompanied by reduced response conservatism. Focusing on task performance, the reduction in RT and omission errors indicates enhanced efficiency in stimulus processing and response execution. These results are consistent with previous evidence indicating that physical activity interventions can enhance cognitive–motor performance in older people, particularly in tasks requiring rapid information processing and response selection (Stillman et al., 2020; Northey et al., 2018). However, the concurrent increase in commission errors suggests that a reduction in inhibitory restraint accompanied these gains. This pattern supports the notion that performance in Go/No-Go paradigms reflects a dynamic balance between response activation and suppression rather than a unitary inhibitory process (Diamond, 2013; Verbruggen & Logan, 2008; Heitz, 2014).

From this perspective, the present findings align with theoretical accounts of aging that emphasize strategic adaptations rather than purely deficit-based interpretations. Older people are known to adopt more conservative response strategies, prioritizing accuracy over speed (Salthouse, 1979; Hardwick et al., 2022). The present results suggest that the intervention may have shifted this balance toward faster responding, potentially reflecting increased confidence, improved processing efficiency, or reduced response caution. Importantly, this shift should not be interpreted as improved inhibitory control, but rather as a modification in response strategy within the speed–accuracy continuum. Accordingly, the present findings are more appropriately interpreted as reflecting changes in Go/No-Go performance and response strategy rather than as improvements in MI.

These findings should also be interpreted in light of the study design. The absence of a control group limits the ability to fully disentangle intervention-related effects from alternative explanations such as repeated testing or task familiarization. Repeated exposure to the Go/No-Go task may have contributed to improvements in RT and omission errors. Therefore, the findings are best interpreted as preliminary within-subject evidence rather than causal effects. Consequently, the observed improvements in cognitive–motor performance cannot be unequivocally attributed to the intervention itself, as they may also reflect nonspecific influences such as repeated testing, task familiarization, or temporal changes. Therefore, the present findings should be interpreted as preliminary within-subject changes associated with the intervention rather than as evidence of intervention efficacy. In addition, the present results differ from previous meta-analytic work indicating limited or inconsistent effects of cognitive training on inhibitory control in older people (Olegário et al., 2023; Dhir et al., 2021). One possible explanation relates to the nature of the intervention. Unlike purely computerized cognitive training, the present program combined physical and cognitive demands within an ecologically relevant context that required continuous perception–action coupling. This integrative approach may preferentially enhance processes such as attentional control, processing speed, and action execution, which indirectly influence performance in inhibition tasks without specifically targeting inhibitory mechanisms.

Beyond task-specific performance, cognitive status, as measured by the MEFO, also improved significantly following the intervention. Given that this instrument captures multiple domains, including memory, phonetic fluency, and temporal–spatial orientation, these findings suggest that the observed changes may reflect broader cognitive engagement beyond task-specific effects. This is consistent with previous evidence indicating that MCT programs can positively influence global cognitive function in older people (Northey et al., 2018; Eggenberger et al., 2015; Liu et al., 2025). In particular, interventions combining physical and cognitively demanding elements have been associated with improvements in executive function and attentional control (Netz et al., 2023), which may contribute to the observed gains in both cognitive and cognitive–motor domains.

In contrast to these objective improvements, HRQoL showed limited responsiveness to the intervention. Improvements were observed only in the general health dimension, while the remaining domains remained stable. This pattern differs from studies reporting broader HRQoL benefits following physical activity interventions in older people (Marquez et al., 2020; Valenzuela et al., 2020). However, this discrepancy may be explained by methodological and baseline factors. High initial scores in several domains, particularly physical role and emotional role, suggest the presence of ceiling effects, which may have limited sensitivity to detect change. Additionally, subjective measures of health perception may require longer intervention periods or more pronounced functional changes to reflect measurable improvements. These findings suggest that objective performance changes may precede subjective improvements in perceived health status. Beyond task-specific performance, modest improvements were observed in global cognitive status, whereas changes in HRQoL were limited and restricted to the general health dimension. These findings suggest that the secondary outcomes should be interpreted cautiously and do not indicate broad improvements in cognition or HRQoL. Ceiling effects may also have influenced the limited changes observed in HRQoL across several SF-36 domains, particularly physical role. Because participants presented relatively high baseline scores in these domains, the opportunity to detect further improvements was reduced. Consequently, the absence of significant changes across most HRQoL dimensions should be interpreted with caution, as it may partly reflect the instrument’s limited ability to detect further improvements among participants with high baseline scores. Future studies may benefit from incorporating alternative HRQoL instruments with greater sensitivity to detect subtle intervention-related changes in relatively healthy older people.

Taken together, these findings indicate domain-specific adaptations, with more pronounced effects in cognitive–motor performance than in subjective health perception. Notably, improvements in RT and cognitive status were not paralleled by improvements in inhibitory accuracy, reinforcing the importance of distinguishing between different components of performance in Go/No-Go tasks. This dissociation highlights that enhanced efficiency in response execution does not necessarily translate into improved response inhibition, particularly in older populations where strategic adjustments play a central role. Although the observed changes were statistically significant, their clinical significance should be interpreted with caution. The reduction in RT may reflect improved processing efficiency during cognitive–motor task performance; however, whether a mean reduction of approximately 51.5 ms translates into meaningful functional benefits in everyday activities remains uncertain. Likewise, the improvement observed in the global MEFO score was modest, and changes in HRQoL were limited to the general health domain. Consequently, these findings should be considered preliminary and interpreted in the context of the present pilot study until confirmed in larger randomized controlled trials evaluating clinically meaningful outcomes. From a mechanistic perspective, the observed effects may be explained by the combined influence of physical and cognitive stimulation. MCT programs are known to induce peripheral and central adaptations, including improvements in cardiovascular function, neural efficiency, and neuroplasticity (Northey et al., 2018; Erickson et al., 2011). The integration of cognitively engaging activities may further potentiate these effects by recruiting executive networks involved in attention, working memory, and action monitoring (Raichlen & Alexander, 2017). These mechanisms are likely to support improvements in processing speed and task engagement, which influence performance on cognitive–motor tasks. Importantly, because the intervention did not specifically target inhibitory control, the observed changes are more plausibly explained by improvements in underlying cognitive–motor processes rather than by direct enhancement of inhibitory capacity. These findings may have implications for the design of multidomain rehabilitation programs targeting cognitive–motor function in aging populations. However, although these mechanisms provide a biologically plausible framework for interpreting the present findings, they were not directly assessed in this study. Therefore, the proposed contributions of neuroplasticity, neural efficiency, cardiovascular adaptations, and executive network recruitment should be considered hypothesis-generating rather than conclusions directly supported by the present data. Future studies that combine multidomain interventions with neurophysiological or neuroimaging measures will be necessary to examine the mechanisms underlying changes in cognitive–motor performance directly.

### 4.1. Limitations and Strengths

This study has several limitations that should be acknowledged. First, the pre-experimental design without a control group limits the ability to establish causal relationships between the intervention and the observed changes. Accordingly, future randomized controlled studies with larger sample sizes, an appropriate control group, and long-term follow-up assessments are warranted to confirm the present findings and assess the durability of the observed changes. Second, the sample size was relatively small, which may reduce statistical power to detect subtle effects, particularly in HRQoL outcomes. Third, ceiling effects in several SF-36 domains restricted variability and may have masked potential improvements. Fourth, the specific contribution of the cognitive stimulation component cannot be isolated, as engagement with these activities was not objectively quantified. Fifth, potential practice or fatigue effects associated with repeated exposure to the Go/No-Go tasks cannot be fully ruled out. Although trial-level RT analyses accounted for repeated observations within participants, trial order was not explicitly modeled; therefore, temporal effects across the continuous sequence of randomized trials cannot be completely excluded. Sixth, the inclusion of only older women limits generalizability to other populations. An additional limitation is that participants’ engagement with the cognitive activities was not objectively quantified. No standardized measures of cognitive engagement or task performance were collected. Future studies should incorporate objective indicators of cognitive engagement to better characterize adherence to the cognitive component of multidomain interventions. Finally, the absence of long-term follow-up limits the ability to draw conclusions regarding the persistence of the observed effects. Despite these limitations, this study provides ecologically valid evidence on the effects of combined physical and cognitive interventions in older women, using a multidimensional assessment of cognitive–motor performance.

### 4.2. Practical Implications

The present findings have several practical implications for interventions targeting cognitive and motor function in older people. Improvements in RT and omission errors suggest that MCT combined with cognitive stimulation may be associated with improved cognitive–motor efficiency, which is critical for everyday tasks requiring rapid decision-making and response execution. The integration of cognitively stimulating activities into rest intervals is a feasible, time-efficient strategy to target both physical and cognitive domains without increasing session duration. This approach may be particularly suitable for community-based interventions aimed at promoting functional independence in older women.

## 5. Conclusions

A 12-week MCT program combined with cognitive stimulation activities was associated with a reorganization of cognitive–motor performance in older women, characterized by faster responses and reduced omission errors alongside increased commission errors. This pattern reflects a shift in the balance between response activation and inhibition rather than a direct improvement in inhibitory capacity. While modest improvements in cognitive status were observed, changes in HRQoL were limited. They should not be interpreted as evidence of broad cognitive or HRQoL benefits. Accordingly, the observed pattern should be interpreted as evidence of a reorganization of Go/No-Go performance and response strategy rather than as an improvement in MI. Future randomized controlled trials with larger sample sizes, long-term follow-up, and neurophysiological measures are warranted to determine the causal effects of combined physical and cognitive interventions and to clarify the biological mechanisms underlying the observed changes in cognitive–motor performance.

## Figures and Tables

**Table 1:** Description of cognitive activities integrated into the multicomponent training program.

	Set	Physical Exercises	Rest Intervention (2 min)	Cognitive Domain
Session A				
Cognitively Stimulating Activities	1	Upper limbs: pull (e.g., standing row, seated row)	Puzzles Part 1	Attention, problem-solving
	2	Lower limbs: knee-dominant (e.g., squats, step-ups)	Puzzles Part 2	Attention, problem-solving
	3	Upper limbs: push (e.g., shoulder press, chest press)	Crossword Part 1	Language, semantic memory
	4	Lower limbs: hip-dominant (e.g., hip thrusts, deadlifts)	Crossword Part 2	Language, semantic memory
	5	Upper limbs: elbow flexion–extension (e.g., barbell curl, triceps extension)	Word games (synonyms, antonyms)	Language, semantic memory
	6	Lower limbs: lower-limb accessory exercises (e.g., calf raises)	Memory card games	Visual memory, attention
Session B				
Cognitive Training	1	Upper limbs: pull (e.g., standing row, seated row)	Rhythmic movement exercises	Coordination, attention
	2	Lower limbs: knee-dominant (e.g., squats, step-ups)	Cross-body coordination tasks	Coordination, attention
	3	Upper limbs: push (e.g., shoulder press, chest press)	Reaction to visual or auditory stimuli	Processing speed, attention
	4	Lower limbs: hip-dominant (e.g., hip thrusts, deadlifts)	Timed tasks	Processing speed, attention
	5	Upper limbs: elbow flexion–extension (e.g., barbell curl, triceps extension)	Repeating sequences	Working memory, attention, inhibitory control
	6	Lower limbs: lower-limb accessory (e.g., calf raises)	Detecting specific stimuli	Processing speed, attention, inhibitory control

**Table 2:** Fixed effects from the linear mixed-effects model examining the effect of session on reaction time.

Fixed Effect	Estimate	SE	df	*t*	*p*
Intercept	522.04	17.41	33.64	29.98	<0.001
Session 2	−30.29	2.94	11,721.6	−10.30	<0.001
Session 3	−51.46	2.94	11,721.6	−17.50	<0.001

Note. Session 1 was used as the reference category. Negative estimates indicate faster reaction times relative to Session 1.

## Data Availability

The raw data supporting the conclusions of this article will be made available by the authors on request.

## Abbreviations

The following abbreviations are used in this manuscript:

BMI	Body mass index
HRQoL	Health-related quality of life
MCT	Multicomponent training
MEFO	Memory, fluency and temporo-spatial orientation
MI	Motor inhibition
RT	Reaction time
